# The Acute Neuromuscular Responses to Cluster Set Resistance Training: A Systematic Review and Meta-Analysis

**DOI:** 10.1007/s40279-019-01172-z

**Published:** 2019-09-11

**Authors:** Christopher Latella, Wei-Peng Teo, Eric J. Drinkwater, Kristina Kendall, G. Gregory Haff

**Affiliations:** 1 Centre for Exercise and Sports Science Research (CESSR), School of Health and Medical Sciences, Edith Cowan University, 270 Joondalup Drive, Joondalup, WA 6027 Australia; 2 Neurophysiology Research Laboratory, School of Medical and Health Sciences, Edith Cowan University, 270 Joondalup Drive, Joondalup, WA 6027 Australia; 3 Physical Education and Sports Science Academic Group, National Institute of Education, Nanyang Technological University, Singapore, Singapore; 4 Institute for Physical Activity and Nutrition (IPAN), School of Exercise and Nutrition Sciences (SENS), Deakin University, Geelong, VIC Australia; 5 Centre for Sport Research (CSR), School of Exercise and Nutrition Science, Deakin University, Geelong, VIC Australia; 6 Directorate of Sport, Exercise and Physiotherapy, University of Salford, Greater Manchester, UK

## Abstract

**Background:**

Cluster sets (CSs) are a popular resistance training (RT) strategy categorised by short rest periods implemented between single or groups of repetitions. However, evidence supporting the effectiveness of CSs on acute intra-session neuromuscular performance is still equivocal.

**Objective:**

The objective of this investigation was to determine the efficacy of a single session of CSs to attenuate losses in force, velocity and power compared to traditional set (TS) training.

**Methods:**

Screening consisted of a systematic search of EMBASE, Google Scholar, PubMed, Scopus and SPORTDiscus. Inclusion criteria were (1) measured one or more of mean/peak force, velocity or power; (2) implemented CSs in comparison to TSs; (3) an acute design, or part thereof; and (4) published in an English-language, peer-reviewed journal. Raw data (mean ± standard deviation) were extracted from included studies and converted into standardised mean differences (SMDs) and ± 95% confidence intervals (CIs).

**Results:**

Twenty-five studies were used to calculate SMD ± 95% CI. Peak (SMD = 0.815, 95% CI 0.105–1.524, *p* = 0.024) and mean (SMD = 0.863, 95% CI 0.319–1.406, *p* = 0.002) velocity, peak (SMD = 0.356, 95% CI 0.057–0.655, *p* = 0.019) and mean (SMD = 0.692, 95% CI 0.395–0.990, *p* < 0.001) power, and peak force (SMD = 0.306, 95% CI − 0.028 to 0.584, *p* = 0.031) favoured CS. Subgroup analyses demonstrated an overall effect for CS across loads (SMD = 0.702, 95% CI 0.548–0.856, *p* < 0.001), included exercises (SMD = 0.664, 95% CI 0.413–0.916, *p* < 0.001), experience levels (SMD = 0.790, 95% CI 0.500–1.080, *p* < 0.001) and CS structures (SMD = 0.731, 95% CI 0.567–0.894, *p* < 0.001) with no difference within subgroups.

**Conclusion:**

CSs are a useful strategy to attenuate the loss in velocity, power and peak force during RT and should be used to maintain neuromuscular performance, especially when kinetic outcomes are emphasised. However, it remains unclear if the benefits translate to improved performance across all RT exercises, between sexes and across the lifespan.

**Electronic supplementary material:**

The online version of this article (10.1007/s40279-019-01172-z) contains supplementary material, which is available to authorized users.

## Key Points

**Table:** 

Cluster set (CS) training is an effective means of attenuating velocity and power loss during a resistance training session.
CSs appear to be most beneficial for moderate- and high-load paradigms where fatigue has the potential to impair performance.
Additional research is needed in order to fully understand the benefits of CSs with additional exercises, between sexes and across the lifespan.

## Introduction

### Background

Resistance training (RT) is a fundamental component of athletic development, with the aim of improving performance and minimising injury risk [1–4]. In particular, the work performed during a RT session provides the necessary stimuli for metabolic, muscular and neuromuscular adaptations to occur and, thus, improve performance over time. Furthermore, it is well-established that specific neuromuscular adaptations occur in response to the training stimuli [5]. As such, the manipulation of mechanical stimuli (e.g. movement velocity and load) is considered to be a key training strategy when focusing on the development of muscular strength and power [6, 7].

In practice, designated training blocks are prescribed to progressively increase physiological stress and, thus, develop specific neuromuscular traits (i.e. hypertrophy, strength or power). Fundamentally, RT prescription has focused on empirically based set and repetition schemes performed in a continuous traditional set (TS) configuration [8, 9], such that during TS training, rest intervals are only implemented after the completion of each set. During the early phase of periodised training, higher-volume hypertrophy-inducing programmes have previously been implemented [7, 10, 11], before progressing to lower-volume, higher-intensity programmes designed to facilitate maximal strength development [10, 12, 13]. During peaking phases, an emphasis on power, i.e. 3–5 repetitions (not to failure), with loads that correspond to 30–80% of 1 repetition maximum (1RM), are employed [14]. However, novel strategies such as cluster sets (CSs) have gathered interest for their proposed ability to maximise neuromuscular adaptations, provide overload, maintain training intensity and minimise overtraining [15, 16]. Although anecdotal evidence dates back to the 1950s, CSs were first reported in the literature by Roll and Omer [17] in 1987 and later popularised by Siff and Verkhoshansky [18]. CSs are based on the principle of implementing short, intra-set rest periods between groups of repetitions [15, 19–21]. For example, a TS approach may consist of 4 × 6 continuous repetitions with typically 1–3 min of inter-set rest, in comparison to a CS comprising 4 × (2 × 3 clusters) with 15–45 s of ‘intra-set’ rest implemented between each cluster in addition to the inter-set rest period [15]. However, this has also extended to inter-repetition rest strategies, whereby a short rest period is implemented after each repetition, rest re-distribution, whereby the total rest time calculated from a TS protocol is interspersed evenly between groups of repetitions, or the rest–pause method [16, 22, 23]. Despite the recent interest in CS paradigms, it remains unclear which method of CS application is superior, with continuing debate over the true definition of a CS.

Despite the growing popularity of CSs, an understanding of the acute performance benefits over a training session remains limited. Emerging evidence has suggested a reduction in fatigue [23–27] and an attenuation of the loss in force, velocity and power with CSs during a RT session [19, 21, 26, 27]. For example, fatigue during a RT session can severely reduce movement kinetics due to a combination of central (neural) and peripheral (muscular) factors [28, 29]. In particular, this may be caused, at least in part, by an increase in blood lactate concentration and reduction of adenosine triphosphate and phosphocreatine stores. Although fatigue was previously thought to be necessary, the benefit of performing RT close to momentary failure (i.e. repetition maximum paradigms) is still debatable for strength adaptation [30] and may be adverse for power development. Ultimately, this fatigue contributes to the reduction in velocity, power and work output, especially when performed to repetition failure [31]. Thus, intra-set rest should, at least in theory, attenuate fatigue development and allow for a (1) maintenance in force and velocity (power); (2) maintenance of training intensity; and (3) greater overall amount of work to be performed [15]. Conversely, there are several studies demonstrating that structuring training into CSs does not influence force, velocity or power output [32–34]. Such discrepancies are likely caused by a lack of methodological consistency between studies (e.g. loading schemes) or variability in the equipment used to capture kinetic data, rendering interpretation within the literature difficult. In particular, it is unclear how factors such as loading intensity, exercise selection and training status are affected by CS. Thus, some conjecture remains about the effectiveness of the CS and its ability to positively impact performance during RT.

Therefore, the aim of this investigation was to collate and analyse the available CS literature investigating acute neuromuscular performance. We have systematically and meta-analytically reviewed the data to (1) determine the acute neuromuscular responses (i.e. strength, power and velocity) following an acute CS session; (2) make a direct comparison to TS training; and (3) investigate potential differences between exercise selection, loading strategy, experience level and CS structure. These findings will provide clarity regarding the effectiveness of CS training to attenuate the loss of force, velocity and power across a RT session. It is intended that the findings will help better inform strength and conditioning professionals on effective programme design to maximise neuromuscular stimuli and inform future research areas within the field.

### Objectives

The aim of this investigation was to systematically review and present the results of a meta-analysis regarding the effects of CS training on acute neuromuscular performance (i.e. force, velocity and power), with moderators consisting of exercise selection, loading intensity, training experience of the individual and CS structure.

## Methods

### Research Question and Registration

This systematic review and meta-analysis conformed to the Preferred Reporting Items for Systematic Reviews and Meta-Analysis (PRISMA) guidelines.

The research questions were defined by the PICOS model in accordance with PRISMA guidelines, as follows:

1. *Population:* Males and females with or without RT experience.
2. *Intervention:* An acute RT session which incorporated a ‘CS’ design.
3. *Comparator:* Acute neuromuscular responses compared to TS.
4. *Outcomes:* Peak and/or average force, velocity and/or power.
5. *Study design:* Randomised controlled designs, counterbalanced crossover or repeated measure designs that investigated the acute mechanical/neuromuscular responses from CS training.

### Literature Search

Searches for this review were performed using the EMBASE, Google Scholar, PubMed, Scopus and SPORTDiscus electronic databases without any year restriction. The following words were combined and used for the searches through article title, abstract and keyword screening: (‘cluster-set*’ OR ‘cluster loading’ OR ‘cluster-type’ OR ‘inter-set rest’ OR ‘rest redistribution’ OR ‘rest-loading’ OR ‘rest-pause’ OR ‘traditional set’ OR ‘intra set’ OR ‘inter rep*’ OR ‘work-to-rest ratio’) AND (‘power’ OR ‘strength’ OR ‘displacement’ OR ‘neur*’ OR ‘repetition’ OR ‘velocity’ OR ‘endurance’ OR ‘performance’ OR ‘volume’ OR ‘work’ OR ‘hypertroph*’ OR ‘fatigue’ OR ‘force’ OR ‘perceived exertion’). After the removal of duplicates, the title and abstract of each article was initially screened for suitability. Full-text articles were retrieved in order to determine inclusion or exclusion. In each full text, the reference lists were screened for additional articles. In addition, the list of articles that cited the included studies (i.e. forward citation tracking) were screened. Two authors (CL and GH) performed the search independently. In the case of any selection bias, a third assessor (W-PT) was included. The search was conducted throughout September of 2017 and updated in August of 2018.

### Dependent Variables

Dependent variables were grouped into force (maximal/peak and/or average from isometric or dynamic movements), velocity (maximal/peak and/or average of the movement, bar speed or body during acceleration) and power (maximal/peak and/or average calculated in watts, or determined from jump performance).

### Inclusion and Exclusion Criteria

Studies were included in this review if they met the following criteria: (1) measured one or more of peak or average force, power and velocity; (2) implemented CS in comparison to TS; (3) the study had an acute design or part thereof; and (4) was published in an English-language peer-reviewed journal. Data (mean ± standard deviation [SD]) from studies that only reported the results in graphical form were extracted using plot digitising software (PlotDigitizer; https://automeris.io/WebPlotDigitizer/). If this method was not suitable, the author(s) of the studies were contacted to obtain original raw data and subsequently excluded if sufficient data for the analysis of the standardised mean difference (SMD) was unavailable or the authors could not be contacted. Articles that did not include a TS condition as a comparator were also excluded from the analysis.

### Data Extraction

For all included articles, the following data were extracted: (1) study characteristics (author, year, sample size and study design); (2) participant demographics (age, sex and RT experience); (3) RT protocols (CS and TS structure [i.e. rest period, repetitions, number of sets, CS configuration, exercise selection and intensity]); and (4) outcome measures (maximal/peak and/or average force, velocity and power). Quantitative data (mean and SD) from pre- and post-training session, first and last repetition or, where necessary, first and last set were extracted from text, tables and figures if required. Where multiple post-training timepoints were reported, the timepoint immediately following the RT session was used. Where the standard error was reported, this was converted post hoc to SD. To increase reliability, data were extracted by two independent assessors (CL and GGH), and in the case of a discrepancy a third assessor (KK) was used as a moderator.

### Statistical Analysis

As systematic influences and random errors were predicted to be present between study-level ES, random effects meta-analyses were conducted for each of performance variables (i.e., force, velocity and power). All performance variable outcomes were presented as averaged SMD ± 95% confidence interval (CI) values. For each study, SMD was computed such that positive values indicate that the intervention group (i.e. CS training) was superior to the control group (i.e. TS training) [35]. Subgroup analyses were agreed upon a priori to assess the influence of moderator variables of RT on physical performance. Where studies had more than one outcome measure in a particular subgroup, they were combined into a single effect size for analysis [36]. This was done to limit the risk of bias of the aggregated effect of comparing the same dataset within the same meta-analysis. Moderator variables in this study included the following:

1. *Training load*: power (optimal load determined for power development regardless of relative value to 1RM), and low (≤ 60% 1RM), moderate (60–79% 1RM) or heavy (defined as either ≥ 80% 1RM or ≥ 6RM load), irrespective of optimal load for power development.
2. *Exercise type*: strength training (compound or isolated task) versus weightlifting (WL) versus strength + WL versus power.
3. *Training experience*: athletic (State-level or above athletes) versus experienced (> 12 months’ RT experience or could squat 1.5 × body weight) versus recreational (physically active and/or < 12 months’ RT experience).
4. *CS structure*: inter-repetition rest versus intra-set rest versus rest–pause.

**Fig. 1:**
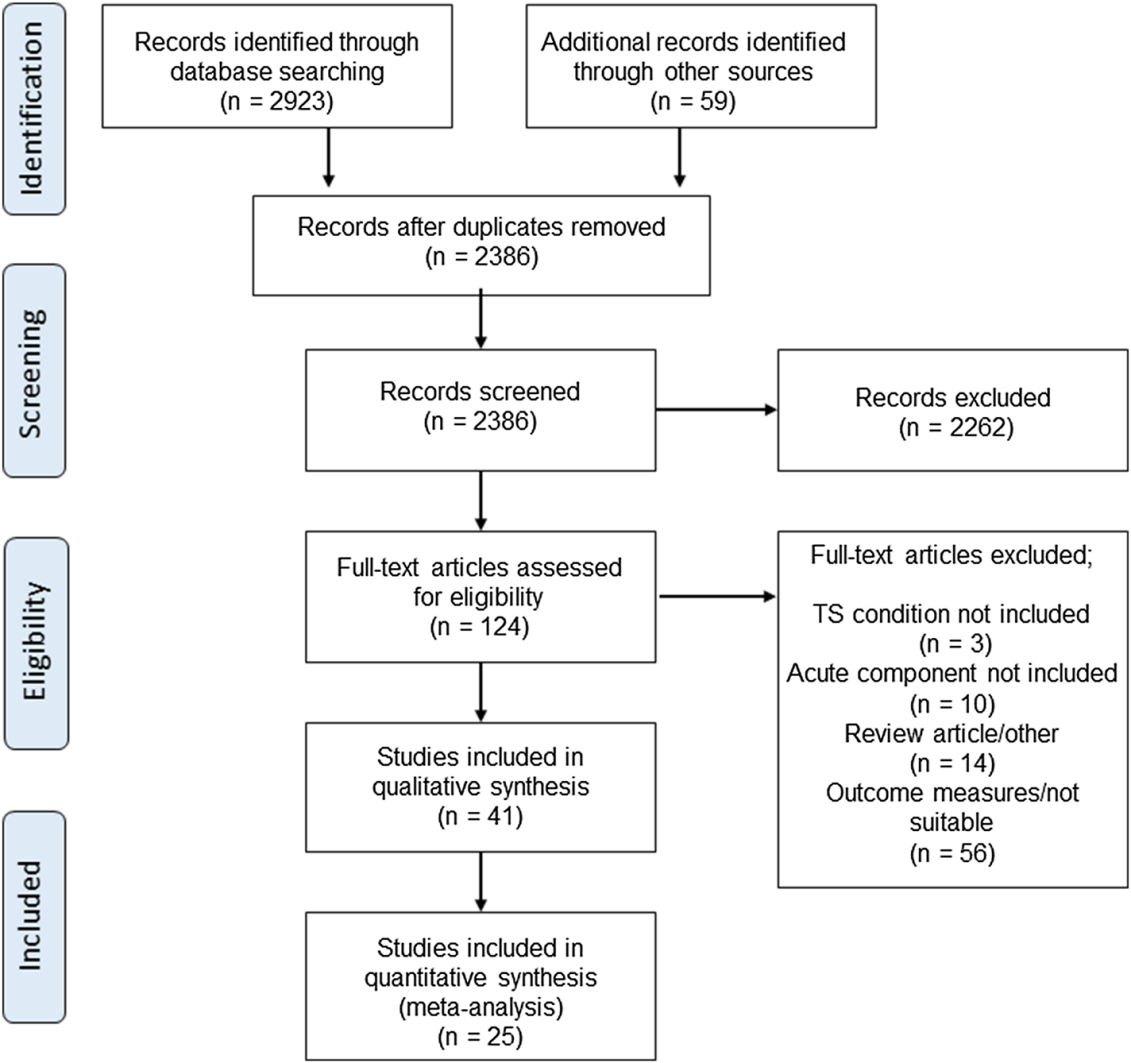
Preferred Reporting Items for Systematic Reviews and Meta-Analysis (PRISMA) flowchart of literature search strategy. *TS* traditional set

Heterogeneity was measured using the *I*^2^ statistic, which indicates the percentage of variance between studies, with cutoff points corresponding to low (0–25%), moderate (26–50%) and high (51–100%) heterogeneity [37]. Funnel plots were used to assess publication bias using Egger’s regression tests where non-significant asymmetry indicated no bias [38] (Electronic Supplementary Material [ESM] Figure 1). All statistical analyses were performed using Comprehensive Meta-Analysis (version 3.0; Biostat, Englewood, NJ, USA). An *α* level of *p* < 0.05 was used to determine statistical significance.

### Methodological Quality and Bias

The methodological quality for each study was evaluated using a modified 11-point Physiotherapy Evidence Database (PEDro) scale; the quality of each study was assessed independently by two authors (CL and KK). Given that it is not possible to blind the participants and investigators in supervised exercise interventions, items 5–7 from the scale, which are specific to blinding, were removed. This approach has been used in previous systematic reviews in the area of RT [39, 40]. With the removal of these items, the maximum result on the modified ‘PEDro 8-point’ scale was 7 because the first item, related to eligibility criteria, is not included in the total score. The qualitative methodology ratings were adjusted similarly to those used in previous exercise-related systematic reviews [39, 40] and were as follows: 6–7 = ‘excellent’; 5 = ‘good’; 4 = ‘moderate’; and 0–3 = ‘poor’. Two assessors (CL and KK) also assessed the bias of included studies using the Cochrane risk of bias assessment tool [35]. The Cochrane risk of bias tool evaluates each study based on the following criteria: sequence allocation, allocation concealment, blinding, incomplete outcome data, selective outcome reporting and other sources of bias. A third reviewer (GGH) acted a moderator if there were discrepancies in the interpretation of the PEDro or Cochrane risk of bias scales.

## Results

### Search Results

**Fig. 2:**
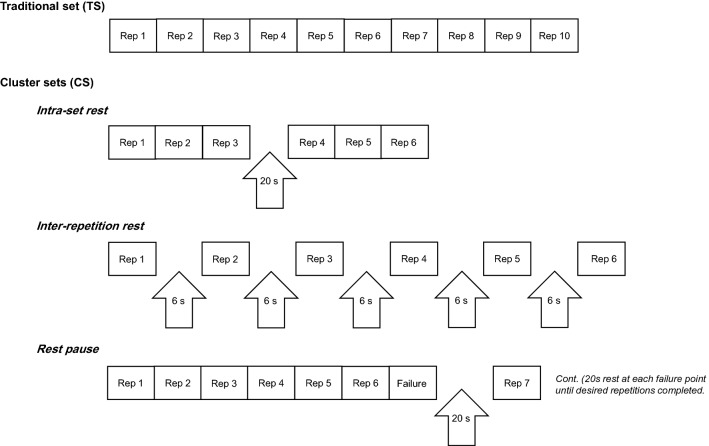
An example of each of the general resistance training paradigms (traditional sets and cluster sets) used in the literature. *Cont.* continue, *Rep* repetition

The search and screening process is presented as a flowchart in Fig. 1. The initial search identified 2923 potentially relevant articles, with 2386 remaining after the removal of duplicates. An additional 2262 articles were excluded following title and abstract screening, and 124 full-text articles were then assessed for eligibility. Based on the selection criteria, a total of 25 were included in the meta-analysis with a total participant sample size of *n = *317. General examples of the TS and CS paradigms employed in the literature can be found in Fig. 2.

### Methodological Quality and Bias

The PEDro scores for the studies in this review ranged from 5 to 6 (mean = 5.7 ± 0.5) (ESM Table 1). Therefore, this result indicates that the evidence used in this review comes from studies with a ‘good’ methodological quality. The Cochrane risk of bias scores indicate a low risk of bias for four of the seven domains (ESM Table 2). Given that allocation concealment, blinding of participants/personnel and outcomes was not feasible in the included studies, we conclude that the generally low risk of bias does not seriously alter the results within or between studies.

### Meta-Analytical Results

A summary of the methods and findings from individual studies is shown in Table 1.

**Table 1:** Summary of the methods and characteristics from the included studies

Study	Sex (sample size)	Age (years) [mean ± SD]	Experience	Resistance exercise(s)	TS and CS structure	CS rest interval(s)	Loading scheme(s)	Outcome measure(s)
Inter-repetition rest								
Boullosa et al. [41]	Male (*n* = 12)	25.5 ± 4.9	Recreational	Half-squat	TS: 1 × 5RM CS: 1 × (5 × 1)	30 s	Load equal to 5RM (heavy)	CMJ, peak power/force
Haff et al. [19]	Male (*n* = 13)	23.4 ± 4.0	Athletic	Clean pulls	TS: 1 × 5 repetitions CS1: 1 × 5 repetitions CS2: 1 × 5 repetitions	30 s	~ 90% or ~ 120% of power clean 1RM (heavy)	Peak power
Hardee et al. [21]	Male (*n* = 10)	23.4 ± 0.4	Experienced	Power clean	TS: 3 × 6 repetitions. CS: 3 × (6 × 1)	20 or 40 s	80% of 1RM (heavy)	Peak force/velocity/power
Lawton et al. [22]	Male (*n* = 26)	18.0 ± 0.3	Athletic	Bench press	TS: 1 × 6 repetitions CS1: 1 × (6 × 1) CS2: 1 × (3 × 2) CS3: 1 × (2 × 3)	23, 56 or 109 s	Load equal to 6RM (heavy)	Mean power
García-Ramos et al. [42]	Male (*n* = 16)	33.7 ± 4.1	Recreational	Half-squat	TS: 6 × sets to failure or 20 repetitions for each load CS: As above	6 s	15% below optimal (low) Load producing maximal power (power) 15% above optimal (heavy)	Peak power Mean power
Iglesias-Soler et al. [43]	Male (*n* = 9)	23.8 ± 4.1	Experienced	Back-squat	TS: 3 × sets to failure CS: 3 × (1 until failure)	45.4 s	Load equal to 4RM (heavy)	Mean velocity
Moir et al. [45]	Male (*n* = 11)	21.9 ± 1.0	Recreational	Deadlift	TS: 1 × 4 repetitions CS1: 1 × (2 × 2) CS2: 1 × (4 × 1)	30 s	Load equal to 90% of 1RM (heavy)	Mean power/force
Wagle et al. [49]	Male (*n* = 11)	26.1 ± 4.1	Experienced	Back-squat	TS: 3 × 5 repetitions CS1: 3 × (5 × 1) CS2: 3 × (5 × 1) with eccentric overload	30 s	80% of 1RM (concentric) 105% of concentric 1RM (eccentric) (heavy)	Mean power/velocity Peak force/power/velocity
Mora-Custodio et al. [50]	Male (*n* = 10)	22.8 ± 3.1	Recreational	Back-squat	TS: 3 × 6, 5, 4 or 3 repetitions CS: rest between each repetition for each set configuration	10 or 20 s	60–80% of 1RM (moderate and heavy)	Mean velocity
Iglesias-Soler et al. [55]	Male (*n* = 10)	23.0 ± 4.0	Experienced	Back-squat	TS: 3 × sets until failure CS: 3 × (1 until failure)	Rest between each repetition with the inter-repetition interval calculated from the TS session	Load equal to 4RM (heavy)	Mean velocity Maximum force
Mayo et al. [56]	Male (*n* = 7), female (*n* = 1)	23.8 ± 1.4	Recreational	Bench-press and squat	TS: 5 × sets to failure CS: 5 × 1 until volume equaled TS condition	Bench press: 24.7 s Squat: 21.9 s	Load equal to 10RM (moderate)	Mean velocity
García-Ramos et al. [57]	Male (*n* = 34)	21.5 ± 2.8	Recreational	Bench-press throw	TS: 1 × 15 repetitions CS1–CS2: 1 × (15 × 1)	6 or 12 s	30–50% of 1RM (power)	Peak velocity
García-Ramos et al. [58]	Male (*n* = 10)	29.4 ± 3.5	Experienced	Smith machine bench-press	TS1: 3 × 10 repetitions TS2: 6 × 5 repetitions CS1–CS3: 3 × (10 × 1)	5, 10 or 15 s	75% of 1RM (moderate)	Mean velocity
Nickerson et al. [52]	Male (*n* = 12)	21.0 ± 2.0	Athletic	Back-squat	TS: 1 × 3 repetitions CS: 1 (3 × 1)	30 or 60 s	85% of 1RM (heavy)	Mean velocity
Nickerson et al. [59]	Male (*n* = 12)	22.0 ± 3.0	Recreational	Back-squat (with or without elastic bands)	TS: 1 × 3 repetitions CS: 1 (3 × 1)	30 s	85% of 1RM (heavy)	CMJ (peak power)
Intra-set rest								
Oliver et al. [23]	Male (*n* = 12)	25.0 ± 1.0	Experienced	Back-squat	TS: 4 × 10 repetitions CS: 4 × (2 × 5)	30 s	70% of 1RM (moderate)	Mean power
Joy et al. [24]	Male (*n* = 9)	23.0 ± 2.4	Experienced	Back-squat	TS: 4 × 10 repetitions CS: 4 × (2 × 5)	60 s	75% of 1RM (moderate)	Mean power
Tufano et al. [25]	Male (*n* = 12)	25.8 ± 5.1	Experienced	Back-squat	TS: 3 × 12 repetitions CS1: 3 × (3 × 4) CS2: 3 × (6 × 2)	30 s	60% of 1RM (moderate)	Mean force/velocity/power Peak force/velocity/power
Girman et al. [44]	Male (*n* = 11)	22.9 ± 2.6	Experienced	Multiple	TS: 4 × 6 repetitions and 5 × 4–10 repetitions CS: 4 × (3 × 2) and 5 × (2–5 × 2)	15 s	50–75% of 1RM (moderate)	CMJ Long jump
Oliver et al. [46]	Male (*n* = 10)	27.0 ± 4.0	Experienced	Back-squat	TS: 4 × 10 repetitions CS: 4 × (2 × 5)	30 s	70% of 1RM (moderate)	Mean force/velocity/power
Oliver et al. [47]	Male (*n* = 12)	25.0 ± 1.0	Experienced untrained	Back-squat	TS: 4 × 10 repetitions CS: 4 × (2 × 5)	30 s	70% of 1RM (moderate)	Mean force/velocity/power
Tufano et al. [48]	Male (*n* = 12)	26.0 ± 4.2	Experienced	Back-squat	TS: 3 × 12 repetitions× CS1: 3 × (3 × 4) CS2: 3 × (6 × 2)	30 s	TS: 60% of 1RM CS1: 75% of 1RM (moderate) CS2: 80% of 1RM (heavy)	Mean force/velocity/power Peak force/velocity/power
Koefoed et al. [51]	Male (*n* = 8), female (*n* = 2)	26.5 ± 4.8	Recreational	Jump squat	TS: 4 × 6 repetitions CS: 4 × (3 × 2)	20 s	40% of body mass (power)	Peak force/velocity/power CMJ
Rio-Rodriguez et al. [54]	Male (*n* = 11)	21.0 ± 2.0	Recreational	Knee extensions	4 × sets until 80% of the time to task failure 16 × sets until 20% of time to task failure	36 s	Load corresponding to 50% of maximal voluntary contraction (low)	Maximum force
Rest–pause								
Marshall et al. [53]	Male (*n* = 14)	25.0 ± 1.7	Experienced	Back-squat	TS: 4 × 5 repetitions CS: initial set to failure, 20 s between subsequent sets	20 s	80% of 1RM (heavy)	Maximum force

*CMJ* countermovement jump, *CS* cluster set, *RM* repetition maximum, *SD* standard deviation, *TS* traditional set

#### Kinetic Variables

**Fig. 3:**
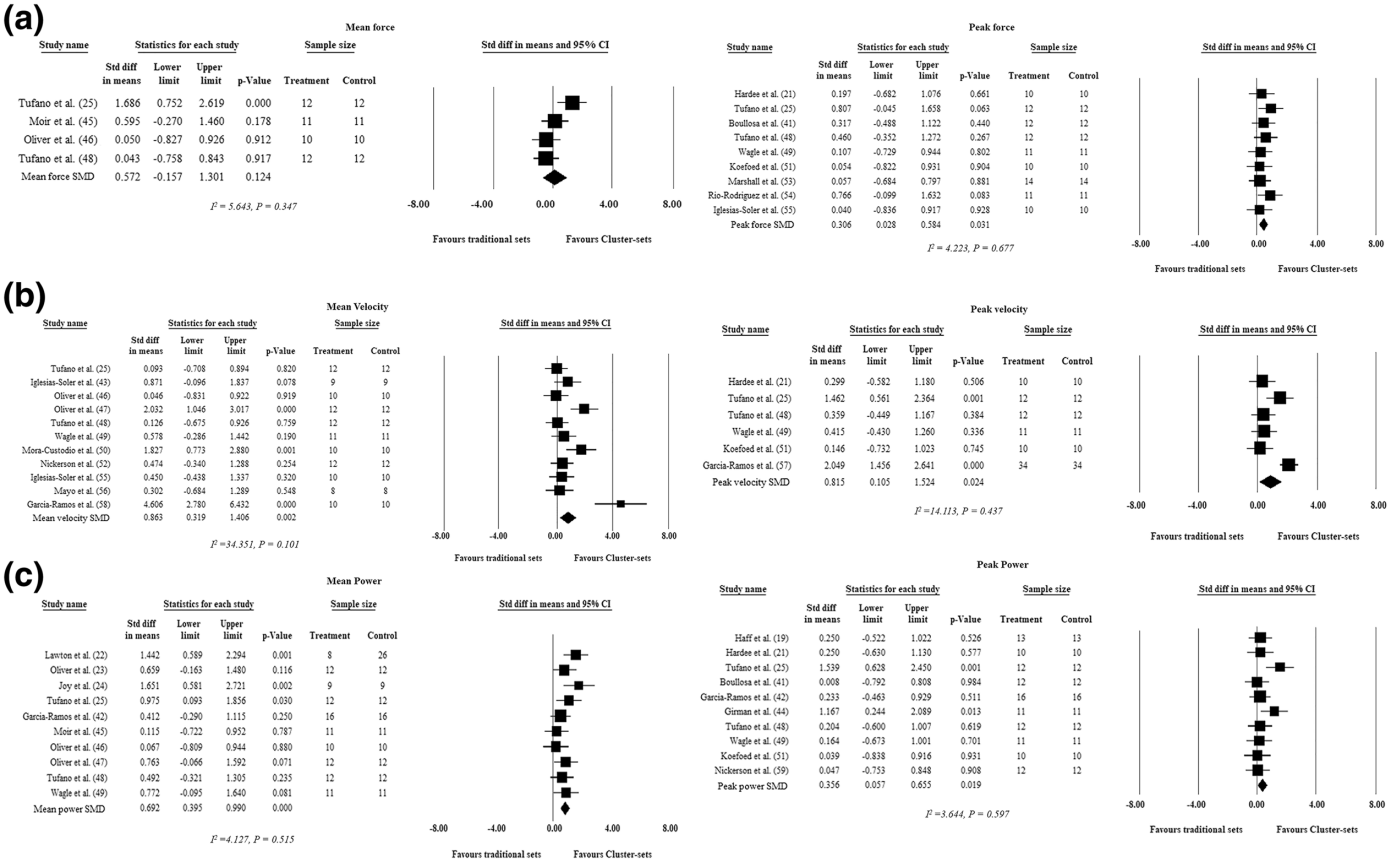
Standardised mean difference, upper and lower confidence limit (95% confidence interval), and *p* value of each individual study and overall effect for **a** mean and peak force, **b** mean and peak velocity, and **c** mean and peak power. Significance indicated by *p *< 0.05. No difference in kinetic variables (i.e. between force, power and velocity) were observed between traditional set and cluster set training. *CI* confidence interval, *diff* difference, *SMD* standardised mean difference, *Std* standard

Power was the most assessed outcome (16 individual studies, *n* = 181 individuals) (peak power: SMD = 0.356, 95% CI 0.057–0.655, *p* = 0.019; mean power: SMD = 0.692, 95% CI 0.395–0.990, *p* < 0.001) [19, 21–25, 41, 42, 44–49, 51, 59], followed by velocity (14 individual studies, *n* = 170 individuals) (peak velocity: SMD = 0.815, 95% CI 0.105–1.524, *p* = 0.024; mean velocity: SMD = 0.863, 95% CI 0.319–1.406, *p* = 0.002) [21, 25, 43, 46–52, 55–58] and then force (11 individual studies, *n* = 123 individuals) (peak force: SMD = 0.306, 95% CI − 0.028 to 0.584, *p* = 0.031; mean force: SMD = 0.572, 95% CI − 0.157 to 1.301, *p* = 0.124) [21, 25, 41, 45, 46, 48, 49, 51, 53–55]. The individual study, subgroup analyses and overall SMD ± 95% CI for kinetic variables can be found in Fig. 3a–c.

#### Exercise Selection

**Fig. 4:**
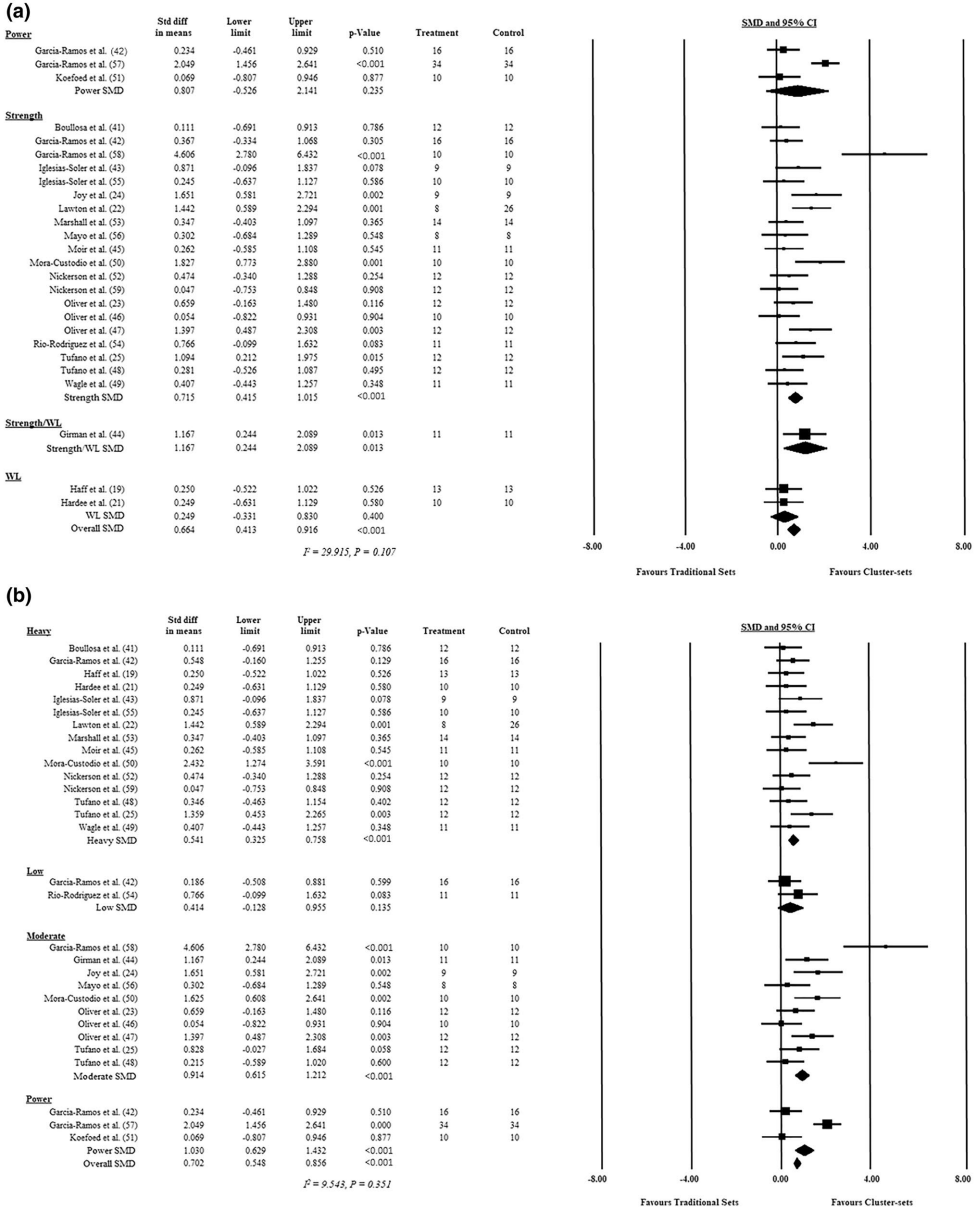

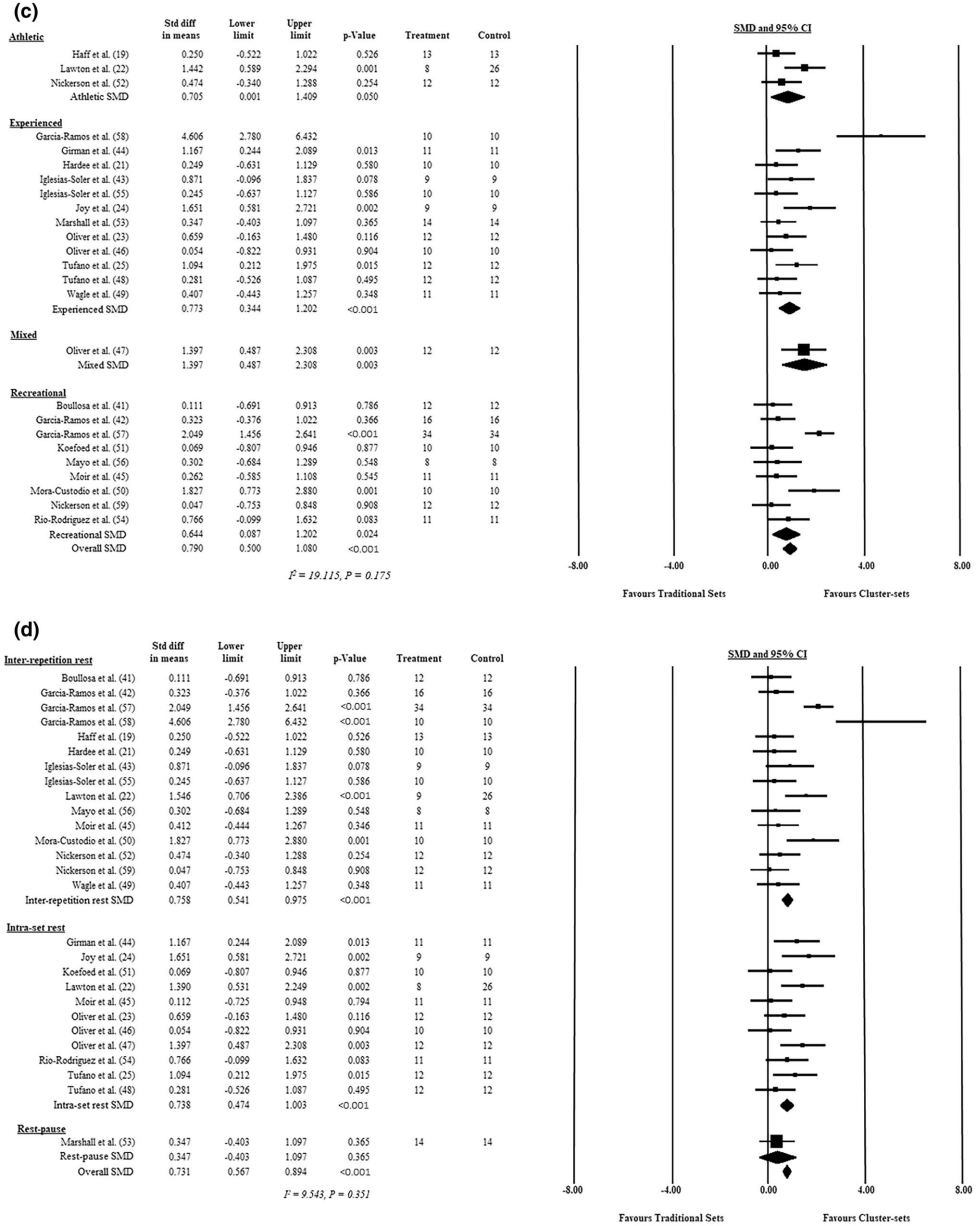
Standardised mean difference, upper and lower confidence limit (95% confidence interval), and *p* value of each individual study and overall effect for **a** exercise type, **b** loading strategy, **c** resistance training experience and **d** cluster set protocol. Significance indicated by *p *< 0.05. No differences were observed between outcomes in any subgroup. *CI* confidence interval, *diff* difference, *SMD* standardised mean difference, *Std* standard, *WL* weightlifting

A total of 20 studies included in the meta-analysis used a strength-based exercise, of which 15 used a back squat or half-squat exercise [23–25, 41–43, 46–50, 52, 53, 55, 59], three used the bench press exercise [22, 56, 58], one used the deadlift [45] and one used an isometric knee extension exercise [54]. Two studies assessed a WL task (i.e. clean pulls or power clean) [19, 21], one study used a jump squat (power) [51], one study used the bench press throw [57] and one study combined strength and WL exercises [44]. An overall effect for exercise selection was observed (SMD = 0.664, 95% CI 0.413–0.916, *p* < 0.001), but no differences were detected between strength, WL, power and strength/WL exercises (Q[3] = 2.561, *p* = 0.431). The individual study, subgroup analysis and overall SMD ± 95% CI for exercise selection can be found in Fig. 4.

#### Loading

A total of 15 studies included in the meta-analysis used a heavy loading scheme [19, 21, 22, 25, 41–43, 45, 48–50, 52, 53, 55, 59], ten used a moderate loading scheme [23–25, 44, 46–48, 50, 56, 58], two used a low loading scheme [42, 54] and three used a load considered optimal for power development [42, 51, 57]. It should be noted that three studies used more than one loading scheme [42, 48, 50]. An overall effect for loading intensity was observed (SMD = 0.702, 95% CI 0.548–0.856, *p* < 0.001), but no differences were detected between low, moderate, heavy and power loading schemes (*Q*[3] = 2.376, *p* = 0.301). The individual study, subgroup analysis and overall SMD ± 95% CI for loading intensity can be found in Fig. 4b.

#### Resistance Training (RT) Experience

Twelve studies included in the meta-analysis used experienced individuals [21, 23–26, 43, 44, 46, 49, 53, 55, 58], nine studies used recreational individuals [41, 42, 45, 50, 51, 54, 56, 57, 59], while three used athletic individuals [19, 22, 52]. One study [47] used a combination of recreational and experienced individuals. An overall effect for RT experience was observed (SMD = 0.790, 95% CI 0.500–1.080, *p* < 0.001), but no differences were detected between recreational, experienced, athletic and mixed experience levels (*Q*[3] = 4.008, *p* = 0.332). The individual study, subgroup analysis and overall SMD ± 95% CI for RT experience can be found in Fig. 4c.

#### Cluster Set (CS) Structure

Fifteen studies included in the meta-analysis used the inter-repetition rest method [19, 21, 22, 41–43, 45, 49, 50, 52, 55–59], 11 studies used the intra-set rest method [22–25, 44–48, 51, 54], while only one study used the rest–pause technique [53]. Two studies [22, 45] used both inter-repetition and intra-set rest in their study designs. An overall effect for CS structure was observed (SMD = 0.731, 95% CI 0.567–0.894, *p* < 0.001), but no differences were detected between the inter-repetition rest, intra-set rest and rest–pause method (*Q*[3] = 2.675, *p* = 0.367). The individual study, and overall SMD ± 95% CI for CS structure can be found in Fig. 4d.

## Discussion

This is the first meta-analytical investigation comparing the acute neuromuscular effects of CS versus TS in RT. Specifically, the results of this investigation demonstrate that velocity and power benefit from the use of CS strategies, with the overall magnitude considered statistically significant. Force was not different between CS and TS strategies. Additionally, the benefit of using CS during an acute bout of RT extends across strength and WL tasks, individual experience levels (i.e. recreational, experienced and athletic) and moderate or heavy loading strategies. No differences were observed between subgroup categories. Thus, strength and conditioning professionals should consider using CS as an efficacious strategy during acute RT sessions. Specifically, CS should be used when kinetic variables are emphasised, such as those targeting the optimisation of velocity and power outcomes regardless of training experience.

### Exercise Selection

The use of CS paradigms demonstrated a collective benefit for strength and WL exercises. Given that it is common to utilise a combination of, or all, exercises (e.g. squat, deadlift, bench press and power clean) concurrently during a RT session, and at various stages of a periodised plan, the findings suggest that CS strategies can be used across multiple exercises to optimise acute performance. Moreover, only one study, Rio-Rodriguez et al. [54] used a single joint task. Given programmes emphasising power give precedence to multi-joint movements, implementing CSs for isolated tasks is unlikely to offer the same benefit for athletic performance. Moreover, it is important to note that the majority of evidence stems from lower- or full-body tasks, with only three studies [22, 56, 58] investigating the bench press exercise. Lawton et al. [22] and García-Ramos et al. [58] demonstrated a significant effect (SMD = 1.442, *p* = 0.001 and SMD = 4.606, *p* < 0.001, respectively), despite a non-significant result observed in the study by Mayo et al. [56] (SMD = 0.302, *p* = 0.548). Thus, the limited evidence from upper-body investigations makes it difficult to draw conclusions about the overall effectiveness of CSs between upper- and lower-limb tasks. In particular, some evidence suggests that the development of fatigue [60] and level of perceived exertion [56] differs between the upper and lower limbs. Specifically, Vernillo et al. [60] demonstrated that maximal leg exercise induces a greater magnitude of fatigue, approximately 12% more than an equivalent time-equated upper-body task. Thus, it can be speculated that the CS intra-set rest period required for upper-limb tasks may be different than for lower-limb tasks to maintain or attenuate the loss in performance. For example, Mayo et al. [56] used an inter-repetition rest of 27.4 s, with an improvement observed for the bench press but not back squat exercise when compared to TS. A lower perceived exertion was also reported for the bench press than for squat exercise. Additionally, Lawton et al. [22] demonstrated that mean power was reduced by 53.8 Watts (W), 66.9 W and 57.0 W with inter-repetition rest of 23 s and intra-set rests of 56 s and 109 s, respectively, during a bench press task. Therefore, although the intra-set or inter-repetition rest intervals in the included studies ranged from 6.0 to 45.4 s for lower- and full-body exercises, the lack of a direct comparison to an upper body-specific task limits the generalisation of these findings. Hence, further evidence is required from research investigating upper-limb tasks, which may be particularly important for sports requiring upper-body strength and power to fully understand the benefits of CS training.

### Loading

Intense exercise causes a reduction in neuromuscular performance due to the development of central and peripheral fatigue [28, 29]. Previous evidence has suggested that high-intensity, low-volume exercise causes greater central fatigue, while higher-volume loading schemes cause perturbations at the muscular level [61]. Regardless, the development of fatigue, whether central or peripheral in origin, is considered adverse to the development of force and power due to reductions in neural drive and/or disturbances to intramuscular homeostasis [62, 63]. When grouped by loading intensity, the results of this meta-analysis revealed that CSs were beneficial for optimising acute neuromuscular performance for moderate and heavy loads. Interestingly, despite the known differences in peripheral fatigue development between moderate and heavy load RT schemes [61], no significant effect was found between the included studies. Moreover, the study by García-Ramos et al. [42] demonstrated that CSs were better than TSs across low, high and optimal loads at attenuating power loss. Likewise, the reduction in velocity was less for all loads between 60 and 80% of 1RM for the back squat in the study by Mora-Custodio et al. [50], with a benefit also demonstrated by Tufano et al. [48] using either 75% or 80% of 1RM. This observation warrants some discussion given that the studies utilising moderate loads generally had a higher overall volume/number of performed repetitions [24, 25, 44, 47, 48, 56]. Thus, it could be theorised that the increase in blood lactate concentration and reduction of adenosine triphosphate and phosphocreatine stores [64] as well as alterations in other biomarkers such as cortisol during higher-volume fatiguing TS protocols [65] may be attenuated by CS paradigms. In particular, Haff et al. [20] suggested that the inclusion of short 15–30 s rest intervals may attenuate these changes, which have previously been associated with a reduction in force and velocity during a RT session [6, 66, 67]. However, the results of this meta-analysis do not substantiate these reports. Moreover, it is worth mentioning that it is not clear whether fatigue is required for neuromuscular adaptation to occur [30]. Thus, achieving the same volume load with minimal fatigue development may be a more favourable approach. It should also be noted that biochemical correlates of fatigue were only reported in a handful of the studies [23, 44, 46, 55] examined in this meta-analysis, suggesting that further work in this area is warranted.

Although no significant effect was observed for low load paradigms, this should be interpreted with caution due to the inclusion of only two studies in this subgroup analysis. Although the inclusion of further studies may provide support for CS use with low load paradigms, the results of the study by Rio-Rodriguez et al. [54] require some consideration in itself. Firstly, Rio-Rodriguez et al. [54] used a single-joint isometric knee extension task, which makes it challenging to translate the results of this study to exercises typically used in the preparation of athletes. It should also be noted that the findings from the Rio-Rodriguez et al. [54] study are based on maximal force production and did not consider how the CSs impacted velocity or power. Conversely, although a significant effect was observed for optimal power loading schemes (SMD = 1.030, 95% CI − 0.629 to 1.432), the inclusion of only three studies [42, 51, 57], and the highly significant result from García-Ramos et al. [57], suggests that further research in this area is required before a confident conclusion can be drawn. However, it can be speculated that as power training programmes are not designed to induce large amounts of fatigue, CSs may not be as effective as high-intensity or high-volume protocols that are performed to muscular failure [28, 29, 31].

### RT Experience

CSs offer an additional level of programming complexity by allowing for the manipulation of the rest periods between clusters of repetitions or after each individual repetitions within a set. Furthermore, RT programmes emphasising power development are commonly used for more experienced individuals, or during the later stages of periodised programmes [17]. The results of this meta-analysis did not reveal any significant difference between recreational, experienced and athletic individuals. It should be noted that only three studies used athletic [19, 22, 52] individuals and, likewise, only one study included both recreational and experienced individuals but a subgroup analysis was not reported [47]. However, Oliver et al. [47] made no comparison between experience levels, and thus caution should be used when interpreting these results. Nonetheless, the available evidence suggests that CSs are an efficacious tool for all individuals, regardless of experience, where the emphasis is on maximising kinetic variables during RT.

### CS Structure

As the popularity of CS expands, research continues to investigate the manipulation of the within-set rest periods in an attempt to optimise performance. For example, inter-repetition rest, intra-set rest and the rest–pause method are commonly referred to as a ‘cluster set’. However, the differences in each structure and the subsequent effect on acute neuromuscular performance warrant some discussion.

The results of this meta-analysis revealed a significant benefit for both the inter-repetition rest and intra-set rest CS structures, with less evidence available for the rest–pause method. Specifically, the results of the two studies that included both inter-repetition and intra-set rest in the same investigation [22, 45] did not report any differences between each CS structure. Thus, the evidence from Moir et al. [45], Lawton et al. [22] and the collective evidence presented in this meta-analysis suggests that both inter-repetition and intra-set rest schemes provide an effective means of optimising acute neuromuscular performance. Although no significant effect was observed for the rest–pause method, the fact that only one study, Marshall et al. [53], was able to be included in this subgroup analysis limits the ability to draw confident conclusions regarding this technique. However, as the sets in the study by Marshall et al. [53] were performed until momentary failure, the effectiveness of introducing short rest intervals may be diminished due to accumulated fatigue prior to the implementation of the rest period. Furthermore, initial force and power outputs may differ between set structures (i.e. higher volume vs. lower volume) and, thus, the relative decrease across a set or relative difference between TS and CS should also be considered when interpreting the literature. Therefore, future research investigations are warranted to determine the effectiveness of each CS structure across independent variables (i.e. exercise selection, loading parameters and experience level) in RT.

### Research Recommendations

Given the growing use of CS in applied settings and the gaps highlighted in this meta-analytic review, we suggest several future directions for research in this space. First and foremost, it is clear that there is a paucity of research examining the efficacy of using CS with female cohorts. Although there are known sex differences in the development of exercise induced fatigue [68, 69], it is currently unclear how CSs, which attenuate fatigue development, modulate acute performance in female cohorts. Specifically, given the importance of kinetic variables in athletic performance, distinguishing the effect of CSs on intra-session force, velocity and power characteristics between males and females is warranted. Secondly, the included studies are based on a demographic of young, healthy adults. It has also been established that fatigue differences exist across the lifespan (e.g. fatigue resistance and power development) [70] and, thus, the acute neuromuscular responses to CSs likely differ between the young and old. In particular, power may be of more importance than maximal strength in functional tasks, which likely holds greater relevance in aging populations. For example, recent evidence has supported the use of CS RT interventions to improve functionality in elderly individuals [71]. Furthermore, this review has also highlighted that a relatively large percentage of the evidence stems from lower- or full-body RT exercises, especially the back squat. Thus, future research should also seek to further investigate non-stretch–shorten cycle multi-joint tasks (i.e. deadlift) and applications to strength and power resistance exercises in the upper limbs. Of further interest is that CSs did not have an effect on mean force but may potentially attenuate losses in peak force. However, as suggested in previous work [25, 47], movement velocity, rather than force (especially mean), is considered to be the main factor influencing power output. Based on the available evidence from the literature, we cannot say for certain whether other factors such as a change in impulse or movement strategy (i.e. that which affects range of motion) also contributed at least partly to this observation. Lastly, given biochemical correlates were only investigated in a handful of studies [23, 44, 46, 55], further work should seek to understand the effect of volume-matched TS or CS RT on endocrine and other physiological responses and provide a comprehensive profile of fatigue and subsequent recovery following advanced RT paradigms.

From a methodological perspective, the collective body of evidence comes from studies with ‘good’ methodological quality and a low risk of bias. However, it should be noted that seven of the 25 included studies were not, or did not clearly indicate if the conditions were, randomised. Thus, future research needs to consider the sequence order of trials in order to minimise the potential learning or order effects that can be associated when randomisation is not utilised. Although both items relating to ‘blinding’ suggest a high level of bias, we acknowledge when performing RT studies it is not possible to blind participants or personnel to the treatment being administered and therefore this should not be considered to be confounding factor in the field of research.

## Conclusion

Collectively, the results of this investigation highlight the benefit of CSs to maximise neuromuscular performance during an acute RT session. In particular, the loss of velocity and power, and potentially peak force, can be attenuated via intra-set, inter-repetition and rest–pause paradigms. Given that mean force was not different between CSs and TSs, and power is a function of force and velocity, it seems logical that velocity should be considered in the primary assessment of CS efficacy. Moreover, strength and conditioning professionals should also consider the use of CSs as a tool for maintaining movement velocity across a RT set, or series or sets. Additionally, it is important to consider the impact of the CS design, including intra-set and total repetitions per set, when aiming to maximise velocity and power. Furthermore, when strength and conditioning professionals decide to implement CSs into their athlete training programmes, it is important to realise that these set structures could be beneficial for strength, WL and tasks where moderate and heavy loading schemes are employed. Ultimately, when training to maximise kinetic variables and maintain high-volume loads in a time-efficient manner, CSs can be employed by individuals with a diverse training background ranging from those with minimal to extensive RT experience. While the current research strongly suggests there are positive benefits from employing CS, there is a need for extensive research into the potential differences between the sexes, across the age span and a wider variety of exercises. Finally, future research examining the impact of employing CSs as part of a long-term training programme are warranted to determine if these acute responses translate into long-term performance gains.

## Electronic supplementary material

Below is the link to the electronic supplementary material.

Displays the heterogeneity between studies. (PDF 165 kb)

Modified PEDro scale and methodological quality assessment of the literature. (PDF 179 kb)

Cochrane risk of bias assessment of the literature. (PDF 232 kb)
