# Supplementary material for: The Acute Neuromuscular Responses to Cluster Set Resistance Training: A Systematic Review and Meta-Analysis
Source: Sports Med. 2019 Sep 11;49(12):1861–77. doi: 10.1007/s40279-019-01172-z (PMC6851217; doi:10.1007/s40279-019-01172-z)

The acute neuromuscular responses to cluster-set resistance training: A systematic review and meta-analysis
Short title: Acute responses to cluster-set training

Sports Medicine

Christopher Latella[1], Wei-Peng Teo[2,3], Eric J. Drinkwater[1,4], Kristina Kendall[1], G. Gregory Haff[1,5]

[1] Centre for Exercise and Sports Science Research (CESSR), School of Health and Medical Sciences, Edith Cowan University, Joondalup, Australia

[2] Physical Education and Sports Science Academic Group, National Institute of Education, Nanyang Technological University, Singapore

[3] Institute for Physical Activity and Nutrition (IPAN), School of Exercise and Nutrition Sciences (SENS), Deakin University, Geelong, Australia

[4] Centre for Sport Research (CSR), School of Exercise and Nutrition Science, Deakin University, Geelong, Australia

[5]Directorate of Sport, Exercise and Physiotherapy, University of Salford, Greater Manchester, United Kingdom.

Correspondence:
Dr Christopher Latella
Email: c.latella@ecu.edu.au

Supplementary Figure 1)

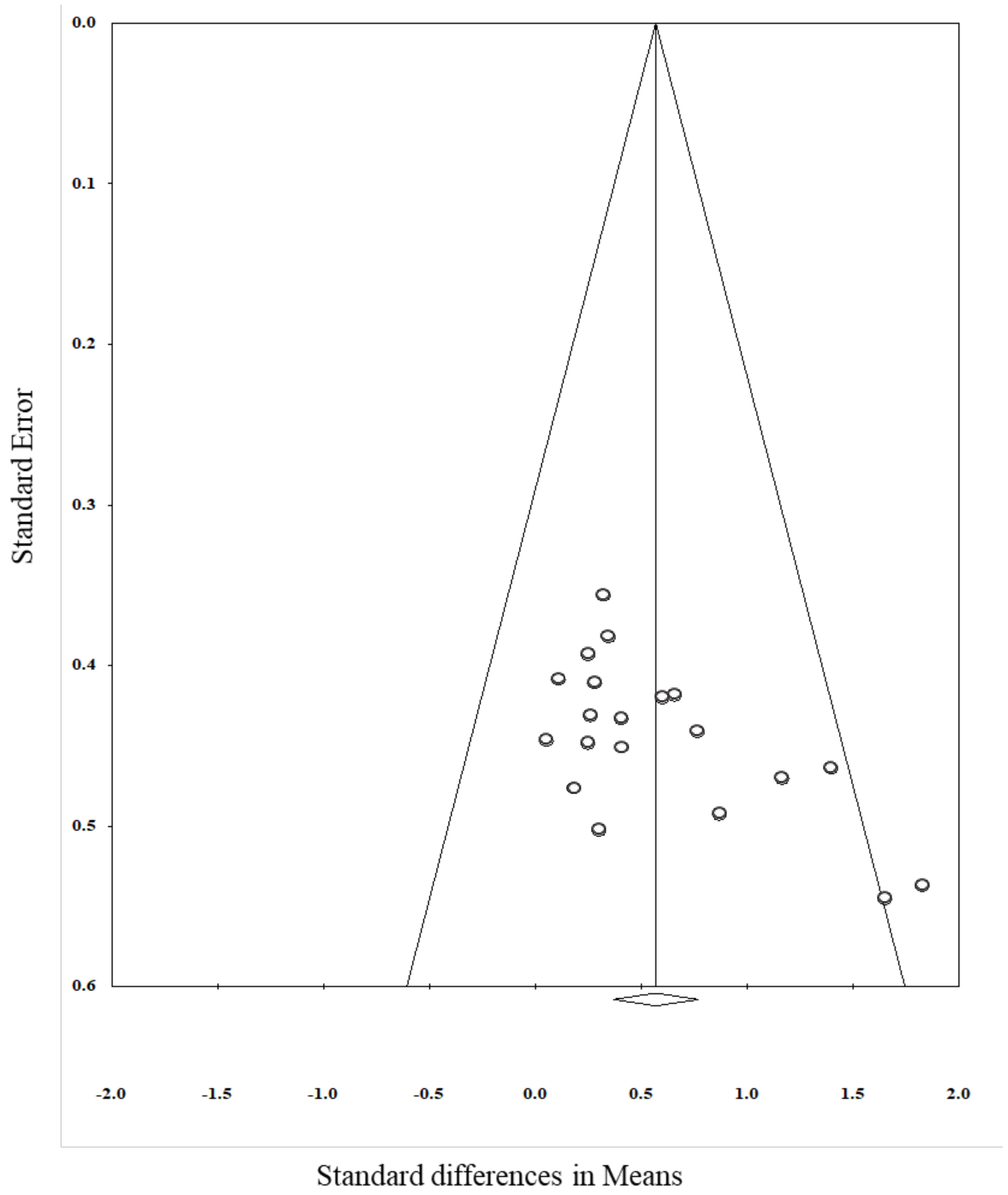

Supplement: Supplementary file 1 — Displays the heterogeneity between studies. (PDF 165 kb) [file 40279_2019_1172_MOESM1_ESM.pdf]
