# Supplementary material for: The Acute Neuromuscular Responses to Cluster Set Resistance Training: A Systematic Review and Meta-Analysis
Source: Sports Med. 2019 Sep 11;49(12):1861–77. doi: 10.1007/s40279-019-01172-z (PMC6851217; doi:10.1007/s40279-019-01172-z)
Supplement: Supplementary file 2 — Modified PEDro scale and methodological quality assessment of the literature. (PDF 179 kb) [file 40279_2019_1172_MOESM2_ESM.pdf]

The acute neuromuscular responses to cluster-set resistance training: A systematic review and meta-analysis
Short title: Acute responses to cluster-set training

Sports Medicine

Christopher Latella[1], Wei-Peng Teo[2,3], Eric J. Drinkwater[1,4], Kristina Kendall[1], G. Gregory Haff[1,5]

[1] Centre for Exercise and Sports Science Research (CESSR), School of Health and Medical Sciences, Edith Cowan University, Joondalup, Australia

[2] Physical Education and Sports Science Academic Group, National Institute of Education, Nanyang Technological University, Singapore

[3] Institute for Physical Activity and Nutrition (IPAN), School of Exercise and Nutrition Sciences (SENS), Deakin University, Geelong, Australia

[4] Centre for Sport Research (CSR), School of Exercise and Nutrition Science, Deakin University, Geelong, Australia

[5]Directorate of Sport, Exercise and Physiotherapy, University of Salford, Greater Manchester, United Kingdom.

Correspondence:
Dr Christopher Latella
Email: c.latella@ecu.edu.au

Supplementary Table 1.

PEDro Assessment Criteria								
Reference (year)	2	3	4	5	6	7	8	Total
Boullosa et al. [41]	Y	N	Y	Y	Y	Y	Y	6
Garcia-Ramos et al. [42]	N	N	Y	Y	Y	Y	Y	5
Garcia-Ramos et al. [57]	Y	N	Y	Y	Y	Y	Y	6
Garcia-Ramos et al. [58]	Y	N	Y	Y	Y	Y	Y	6
Girman et al. [44]	Y	N	Y	Y	Y	Y	Y	6
Haff et al. [19]	Y	N	Y	Y	Y	Y	Y	6
Hardee et al. [21]	Y	N	Y	Y	Y	Y	Y	6
Inglesias-Soler et al. [43]	N	N	Y	Y	Y	Y	Y	5
Joy et al. [24]	Y	N	Y	Y	Y	Y	Y	6
Marshall et al. [53]	Y	N	Y	Y	Y	Y	Y	6
Mayo et al. [56]	N	N	Y	Y	Y	Y	Y	5
Moir et al. [45]	N	N	Y	Y	Y	Y	Y	5
Nickerson et al. [52]	Y	N	Y	Y	Y	Y	Y	6
Nickerson et al. [59]	Y	N	Y	Y	Y	Y	Y	6
Oliver et al. [23]	Y	N	Y	Y	Y	Y	Y	6
Oliver et al. [46]	Y	N	Y	Y	Y	Y	Y	6
Oliver et al. [47]	Y	N	Y	Y	Y	Y	Y	6
Rio-Rodriguez et al. [54]	N	N	Y	Y	Y	Y	Y	5
Tufano et al. [25]	Y	N	Y	Y	Y	Y	Y	6
Tufano et al. [26]	Y	N	Y	Y	Y	Y	Y	6
Mora-Custodio et al. [50]	Y	N	Y	Y	Y	Y	Y	6
Inglesias-Soler et al. [55]	N	N	Y	Y	Y	Y	Y	5
Lawton et al. [22]	Y	N	Y	Y	Y	Y	Y	6
Koefoed et al. [51]	N	N	Y	Y	Y	Y	Y	5
Wagle et al. [49]	Y	N	Y	Y	Y	Y	Y	6

Electronic supplementary material S1 Pedro's scale. y: yes; n: no.

2 = subjects were randomly allocated to groups (in a crossover study, subjects were randomly allocated and order in which treatments were received.

3 = allocation was concealed.

4 = the groups were similar at baseline regarding the most important prognostic indicators.

5 = measures of at least one key outcome were obtained from more than 85% of the subjects initially allocated to groups.

6 = all subjects from whom outcome measures were available received the treatment or control condition as allocated or,

where this was not the case, data for at least one key outcome were analyzed by "intention to treat".

7 = the results of between-group statistical comparisons were reported for at least one key outcome.

8 = the study provided both point measures and measures of variability for at least one key outcome.
