# Supplementary material for: The Acute Neuromuscular Responses to Cluster Set Resistance Training: A Systematic Review and Meta-Analysis
Source: Sports Med. 2019 Sep 11;49(12):1861–77. doi: 10.1007/s40279-019-01172-z (PMC6851217; doi:10.1007/s40279-019-01172-z)
Supplement: Supplementary file 3 — Cochrane risk of bias assessment of the literature. (PDF 232 kb) [file 40279_2019_1172_MOESM3_ESM.pdf]

The acute neuromuscular responses to cluster-set resistance training: A systematic review and meta-analysis
Short title: Acute responses to cluster-set training

Sports Medicine

Christopher Latella[1], Wei-Peng Teo[2,3], Eric J. Drinkwater[1,4], Kristina Kendall[1], G. Gregory Haff[1,5]

[1] Centre for Exercise and Sports Science Research (CESSR), School of Health and Medical Sciences, Edith Cowan University, Joondalup, Australia

[2] Physical Education and Sports Science Academic Group, National Institute of Education, Nanyang Technological University, Singapore

[3] Institute for Physical Activity and Nutrition (IPAN), School of Exercise and Nutrition Sciences (SENS), Deakin University, Geelong, Australia

[4] Centre for Sport Research (CSR), School of Exercise and Nutrition Science, Deakin University, Geelong, Australia

[5]Directorate of Sport, Exercise and Physiotherapy, University of Salford, Greater Manchester, United Kingdom.

Correspondence:
Dr Christopher Latella
Email: c.latella@ecu.edu.au

Supplementary Table 2.

	Risk of bias assessment criteria						
	Sequence generation	Allocation concealment	Blinding of participants, personnel	Blinding of outcome	Incomplete outcome data	Selective outcome reporting	Other source of bias
Boullosa et al. [41]	Low	Unclear	High	High	Low	Low	Low
Garcia-Ramos et al. [42]	High	Unclear	High	High	Low	Low	Low
Garcia-Ramos et al. [57]	Low	Unclear	High	High	Low	Low	Low
Garcia-Ramos et al. [58]	Low	Unclear	High	High	Low	Low	Low
Girman et al. [44]	Low	Unclear	High	High	Low	Low	Low
Haff et al. [19]	Low	Unclear	High	High	Low	Low	Low
Hardee et al. [21]	Low	Unclear	High	High	Low	Low	Low
Iglesias-Soler et al. [43]	High	Unclear	High	High	Low	Low	Low
Joy et al. [24]	Low	Unclear	High	High	Low	Low	Low
Marshall et al. [53]	Low	Unclear	High	High	Low	Low	Low
Mayo et al. [56]	High	Unclear	High	High	Low	Low	Low
Moir et al. [45]	High	Unclear	High	High	Low	Low	Low
Nickerson et al. [52]	Low	Unclear	High	High	Low	Low	Low
Nickerson et al. [59]	Low	Unclear	High	High	Low	Low	Low
Oliver et al. [23]	Low	Unclear	High	High	Low	High	Low
Oliver et al. [46]	Low	Unclear	High	High	Low	High	Low
Oliver et al. [47]	Low	Unclear	High	High	Low	High	Low
Rio-Rodriguez et al. [54]	High	Unclear	High	High	Low	Low	Low
Tufano et al. [25]	Low	Unclear	High	High	Low	Unclear	Low
Tufano et al. [26]	Low	Unclear	High	High	Low	Low	Low
Mora-Custodio et al. [50]	Low	Unclear	High	High	Low	High	Low
Inglesias-Soler et al. [55]	High	Unclear	High	High	Low	Low	Low
Lawton et al. [22]	Low	Unclear	High	High	Low	High	Low
Koefoed et al. [51]	High	Unclear	High	High	Low	Low	Low
Wagle et al. [49]	Low	Unclear	High	High	Low	Low	Low
**Low risk**	**18**	**0**	**0**	**0**	**25**	**19**	**25**
**High risk**	**7**	**0**	**25**	**25**	**0**	**5**	**0**
**Unclear**	**0**	**25**	**0**	**0**	**0**	**1**	**0**
